# Immunogenic recombinant Mayaro virus-like particles present natively assembled glycoprotein

**DOI:** 10.1038/s41541-024-01021-9

**Published:** 2024-12-17

**Authors:** Young Chan Kim, Yasunori Watanabe, Arlen-Celina Lücke, Xiyong Song, Raquel de Oliveira Souza, Robert Stass, Sasha R. Azar, Shannan L. Rossi, Carla Claser, Beate Mareike Kümmerer, Max Crispin, Thomas A. Bowden, Juha T. Huiskonen, Arturo Reyes-Sandoval

**Affiliations:** 1 Department of Paediatrics, Oxford Vaccine Group, University of Oxford, Oxford, UK; 2 Division of Structural Biology, Wellcome Centre for Human Genetics, University of Oxford, Oxford, UK; 3 Institute of Virology, Medical Faculty, University of Bonn, Bonn, Germany; 4 Institute of Biotechnology, Helsinki Institute of Life Science HiLIFE, University of Helsinki, Helsinki, Finland; 5 Department of Parasitology, Institute of Biomedical Sciences, University of São Paulo, São Paulo, SP Brazil; 6 Department of Pathology and the Institute for Human Infection and Immunity, University of Texas Medical Branch, Galveston, TX USA; 7 Department of Microbiology, Immunology and Parasitology, Federal University of São Paulo (UNIFESP), São Paulo, SP Brazil; 8 German Centre for Infection Research (DZIF), Partner Site-Bonn-Cologne, Bonn, Germany; 9 School of Biological Sciences, University of Southampton, Southampton, UK; 10 Instituto Politécnico Nacional, IPN. Av. Luis Enrique Erro s/n. Unidad Adolfo López Mateos, Mexico City, Mexico

**Keywords:** Infectious diseases, Vaccines, Virology

## Abstract

Virus-like particles (VLPs) are an established vaccine platform and can be strong immunogens capable of eliciting both humoral and cellular immune responses against a range of pathogens. Here, we show by cryo-electron microscopy that VLPs of Mayaro virus, which contain envelope glycoproteins E1-E2 and capsid, exhibit an architecture that closely resembles native virus. In contrast to monomeric and soluble envelope 2 (E2) glycoprotein, both VLPs as well as the adenovirus and modified vaccinia virus Ankara (MVA) vaccine platforms expressing the equivalent envelope glycoproteins E1-E2, and capsid induced highly neutralising antibodies after immunisation. The levels of neutralising antibodies elicited by the viral-vectored vaccines of structural proteins and VLPs increased significantly upon boosting. Immunisation of Mayaro virus VLPs in mice with or without an adjuvant (poly:IC) yielded similar levels of neutralising antibodies suggesting that the VLPs may be used for immunisation without the need for an adjuvant. A single or two doses of non-adjuvanted 5 µg of MAYV VLP vaccination provided significant protection against viremia and MAYV-induced foot swelling in the C57BL/6 mouse challenge model. MAYV VLPs represent a non-infectious vaccine candidate, which may constitute a complementary option for future immunisation strategies against this important emerging alphavirus.

## Introduction

Mayaro virus (MAYV) is an emerging, mosquito-borne alphavirus that causes severe chronic arthritis in humans and has the potential to pose a risk in countries where *Aedes spp mosquitoes* are present^1,2^. Chikungunya virus (CHIKV) is endemic in Africa and South/Southeast Asia and has emerged in the Caribbean while MAYV is primarily transmitted by *Haemagogus janthinomys* mosquitoes with outbreaks largely restricted to South America^1,3–7^. However, as MAYV has been isolated from *Aedes spp* mosquitoes, there is growing concern that MAYV could adapt and emerge into urban transmission cycles and *Aedes Albopictus* from Italy was recently shown to be competent vectors for MAYV^8–10^. MAYV could become an important emerging pathogen due to the widespread distribution of *Aedes spp* mosquitoes similar to the recent large-scale epidemics of CHIKV Indian Ocean Lineage (IOL)^2,5,11^. Despite its potential to cause epidemics, there are currently no licensed vaccines or specific treatments against MAYV infections^1^. MAYV belongs to the genus *Alphavirus* within the family *Togaviridae*^12^. MAYV has a single stranded-RNA genome of ~12 kb, which encodes two polyproteins: four non-structural and five structural (s) proteins^13,14^. The non-structural proteins (nsP1, nsP2, nsP3 and nsP4) are required for virus replication^15^. The structural polyproteins (C-E3-E2-6k-E1) are processed to release capsid C, E3, E2, 6k and E1^16^. The viral surface is mainly composed by E1-E2 heterodimers forming spikes^17^, where E1 mediates membrane fusion^18^ and E2 interacts with the host receptor, MXRA8^19,20^ and initiates clathrin-dependent endocytosis^21,22^.

Recent advances in molecular technology have demonstrated the effectiveness of various vaccine platforms especially virus-like particles (VLP) as shown by the successful application of a multivalent human papilloma virus (HPV) vaccine^23^. One of the CHIKV vaccine candidate is a CHIKV VLP based vaccine (VRC-CHIKVLP059-00-VP), which is produced in a mammalian system and has recently completed a phase 2 trial^24^. A randomised, double blind, placebo-controlled, phase 2 trial of the CHIKV VLP vaccine in 400 healthy volunteers from endemic regions demonstrated safety and tolerability with evidence of neutralising antibodies in serum supporting the assessment of efficacy in a phase 3 trial^24^. Two recent Phase 3 trials evaluating an aluminium hydroxide adjuvants CHIKV VLP vaccine have been completed and results demonstrated CHIKV VLP vaccine induced a rapid and robust immune response with a favourable safety profile^25^. These observations support the idea that MAYV VLPs may also serve as a potential vaccine candidate.

MAYV was first reported in 1954 in the Mayaro District of Trinidad and Tobago and continues to emerge and cause disease in Latin America^5,26^. Similar to urban transmission of dengue virus (DENV), CHIKV and Zika virus (ZIKV)^2,5,27,28^, MAYV has the potential to infect millions of people^2,5,28^. However, to date, no MAYV vaccine has entered clinical trials. There are MAYV vaccines that have been shown to be immunogenic in mouse models: an inactivated MAYV vaccine^29^, a live-attenuated MAYV vaccine exhibiting exchange of subgenomic promoter against the internal ribosomal entry site (IRES)^30^, a DNA-based vaccine^31^, a VLP vaccine^32^ and a non-replicating human adenovirus-based vaccine^33^. Additionally, we have recently reported ChAdOx and MVA-based viral-vectored MAYV vaccine candidates^34,35^. ChAdOx1 encoding MAYV structural proteins (ChAdOx1 May) elicited high titres of neutralising antibodies against MAYV and conferred complete protection from a lethal MAYV challenge in a A129 mice model^34^. ChAdOx1 May also provided some degree of cross-protection against a lethal CHIKV challenge^34^. In our previous study, ChAdOx2 May induced high neutralising antibody titres similar to ChAdOx1 May and in addition MVA May was shown to be an effectively boost the antibody response following prime vaccination with ChAdOx1 and ChAdOx2^35^. ChAdOx1 is based on the replication-deficient simian adenoviral vector and it was shown to be well tolerated from safety data compiled from phase I-III clinical trials of ChAdOx1 vectored vaccines including SARS-CoV-2^36^. VLP based vaccines offer a safe and effective alternative to traditional live virus-based vaccines with advantages over other platforms and they have been widely used in the development of vaccines and gene therapy vectors^37,38^. They exhibit a morphology similar to that of the native virus and are safe to use in immunocompromised individuals due to being replication deficient.

Here, we produced MAYV VLPs in mammalian cells using the same codon-optimised sequence of MAYV structural polyprotein (C, E3, E2, 6K, E1: sMAYV) as in our viral vectored vaccines (ChAdOx1/ChAdOx2/MVA)^34,35^. Cryo-EM with single-particle averaging (SPA) was used to confirm that the three-dimensional structure of MAYV VLP closely resembles native virion. Additionally, in-line liquid chromatography-mass spectrometry (LC-MS) confirmed that the glycans presented on E1/E2 on MAYV VLPs are compositionally similar to those presented on other characterised alphaviruses. Sera of mice vaccinated with purified MAYV VLP exhibited high titres of anti-E1 and anti- E2 antibodies, which were shown to be potently neutralising in plaque reduction neutralisation test (PRNT) and VRP-based neutralisation assays. Viral-vectored vaccines and protein sub-unit vaccines were tested alongside the VLPs, which confirmed that MAYV VLP was able to induce high titres of neutralising antibodies comparable to viral-vectored vaccines and significantly higher titres than a E2 sub-unit immunogen. In addition, a single or two doses of non-adjuvanted 5 µg of MAYV VLP vaccination provided significant protection against viremia and MAYV-induced foot swelling in the C57BL/6 mouse challenge model. These observations support further assessment of VLP as a MAYV vaccine candidate.

## Results

### Design of the MAYV structural polyproteins for expression of VLP in mammalian cells

**Fig. 1:**
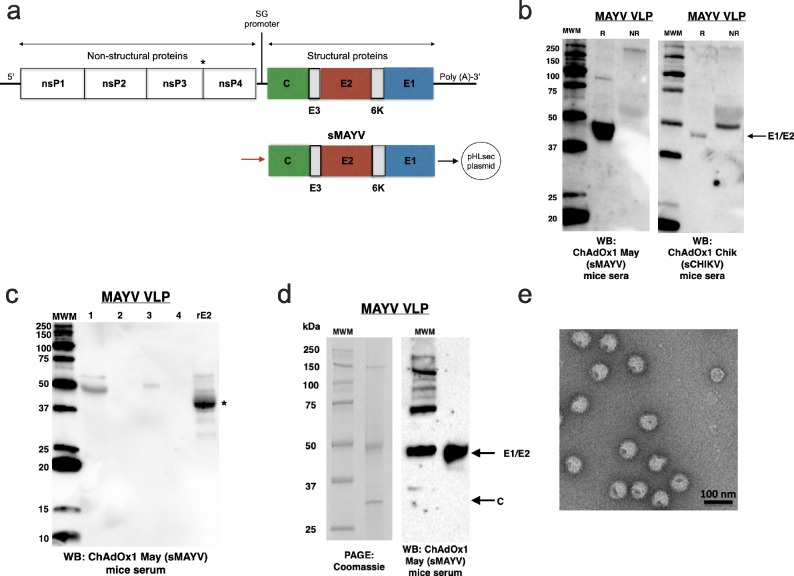
Production, purification and characterisation of MAYV VLP. **a** A schematic representation of the MAYV genome and the structural polyprotein (C, E3, E2, 6K, and E1). **b** Western blot analysis of MAYV VLP production in mammalian cells. The supernatant from HEK293T cells containing VLP was concentrated using 20% sucrose cushion centrifugation prior to WB analysis. WB using mice sera vaccinated with ChAdOx1 May (sMAYV) or Chik (sCHIKV) in reduced (R) and non-reduced (NR) conditions. **c** WB analysis of MAYV VLP following subsequent sucrose gradient purifications compared to recombinant MAYV E2 antigen (without the TM domain) using ChAdOx1 sMAYV. Wells 1 = MAYV VLP after sucrose cushion, 2 = non-infected (mock), 3/4 = MAYV VLP after sucrose gradient top band (3) and bottom band (4), rE2 = recombinant MAYV E2. **d** SDS-PAGE gel (Coomassie staining) and WB of purified MAYV VLP using ChAdOx1 sMAYV vaccinated mice sera under reducing conditions are shown. **e** Transmission electron microscopy (negative stain) images of purified MAYV VLPs are shown.

To test if the genes encoding complete MAYV structural polyprotein (C, E3, E2, 6K, E1: sMAYV) used in our viral vectored vaccines (ChAdOx1 May, ChAdOx2 May and MVA May) can form MAYV VLPs, sMAYV was cloned into the pHLsec expression vector and transfection into mammalian cells was performed^39^ (Fig. 1a). The supernatant from HEK293 cells expressing the sMAYV antigen was collected and concentrated via 20% sucrose cushion. Western blot (WB) analysis of MAYV VLP using mouse sera post immunisation with ChAdOx1 May (sMAYV) showed the presence of the bands around 45 kDa under reducing condition (R), which corresponded to the expected sizes of E1/E2 (Fig. 1b). A same sized band of 45 kDa is detected for MAYV VLPs when ChAdOx1 Chik (sCHIKV) mouse sera was used, which suggests that there is some degree of cross-reactivity between MAYV VLPs and the antibodies produced post ChAdOx1 sCHIKV vaccination (Fig. 1b). To obtain higher purity of MAYV VLPs, this sample was further purified by sucrose gradient and further WB analysis of MAYV VLPs was performed compared to recombinant MAYV E2 antigen without the transmembrane (TM) domain using mouse sera post immunisation with ChAdOx1 sMAYV, which showed the specific band ~50 kDa at the top band of sucrose gradient indicating MAYV VLP and specific band ~40 kDa corresponding to recombinant MAYV E2 (Fig. 1c). To obtain higher concentration of MAYV VLPs for structural study, the supernatant containing the VLPs were PEG precipitated before the ultracentrifugation by sucrose gradient and sucrose cushion and the purified VLPs were verified using SDS-PAGE (Coomassie staining) and WB (Fig. 1d). The integrity of MAYV VLPs was checked by negative stain transmission electron microscopy which revealed uniform particles of ~700 Å (70 nm) in size, which resembled wild-type MAYV particles (Fig. 1e).

### Cryo-EM reconstruction of MAYV VLP

Mature alphaviruses have a T = 4 icosahedral symmetry and consist of 80 envelope trimers of E1-E2 heterodimers (60 quasi-three-fold symmetry trimers and 20 icosahedral three-fold symmetry trimers) and 240 copies of capsid protein^40–42^. The ectodomain structure of the E2-E1 heterodimers of related alphaviruses Semliki Forest virus, Sindbis virus and CHIKV have been studied by X-ray crystallography^18,43–45^. In addition, cryo-EM was used to determine the complete structures of several alphaviruses^41,46–51^, including MAYV virions^52^ but no structure of MAYV VLPs has been published.

**Fig. 2:**
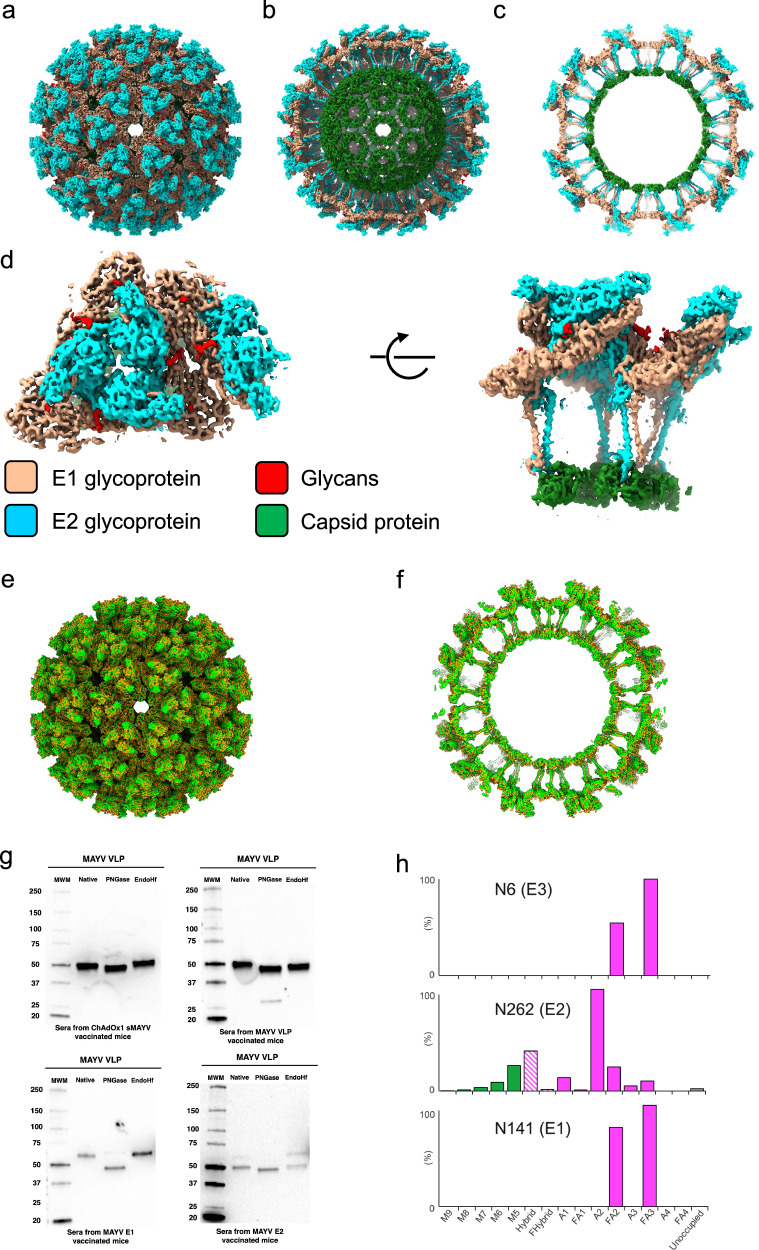
Cryo-EM structure of MAYV VLP to 4.5 Å resolution and compositional analysis of N-linked glycosylation presented by MAYV VLP glycoproteins. **a** Structure of MAYV-VLP seen along the two-fold axis of icosahedral symmetry. **b** The same view as in (**a**), with frontmost E1/E2 glycoprotein spikes removed to reveal the capsid. **c** A central slab of density is shown. **d** One asymmetric unit is shown orthogonally. The symmetric unit is shown rotated 90 degrees around the axis indicated. **e** The structural comparison of our MAYV VLP coloured in lime to mature live MAYV virion in orange (EMDB-22961) and its cross-section display (**f**). (**g**) N-linked glycosylation of purified MAYV VLP produced in HEK293T cells was assessed by WB under reducing condition using mice sera vaccinated with ChAdOx1 sMAYV, VLP, E1 and E2 following digestion with PNGase F or Endo-Hf. **h** N-glycan analysis by in-line liquid chromatography-mass spectrometry of glycopeptides. Green—oligomannose-type glycan, pink—complex-type glycan, pink = hybrid type glycans. Complex glycans are grouped according to the number of antennae and presence of core fucosylation (A1 to FA4).

In this work, cryo-EM structure of MAYV VLP was determined by SPA and icosahedral reconstruction to 4.5 Å resolution (Fig. 2). The MAYV VLP structure is highly similar to the previously published MAYV virion structure^52^, showing the characteristic T = 4 arrangement of the 80 glycoprotein trimers made of E1 and E2 glycoproteins (Fig. 2a) in addition to the capsid protein layer (Fig. 2b, c, e). Additional densities are present corresponding to glycans in positions of E1 N141 and E2 N262 glycosylation sites similar to that of MAYV virion structure reported earlier (Fig. 2d, e)^52^. The structural comparison of our MAYV VLP to this mature live MAYV virion (EMDB-22961) and its cross-section display (Fig. 2e, f) confirms that that our MAYV VLP structure is consistent with the native virus.

### N-glycosylation status of MAYV VLP

To assess if the glycosylation presented by the MAYV VLP and to assess whether the antigenic surface presented by the VLPs resembles that of the native virion, the glycosylation status of MAYV VLPs was analysed by LC-MS. The MAYV E1 and E2 proteins each were predicted to encode an N-linked glycosylation sequon (NXT/S, where X ≠ P) at N141 and N262, respectively, by GlycoEP^53^. In addition, E3 encodes N-linked sequons at N6 and N53, but both N6 and N53 had a low probability of being occupied by GlycoEP^53^.

To analyse the N-glycosylation status of MAYV VLPs, a series of Western blot analyses were performed on purified MAYV VLP, before and after de-glycosylation, using mouse sera vaccinated with ChAdOx1 sMAYV, VLP, E1 and E2 (Fig. 2g). Similar analysis on E3 was not possible due to the lack of anti-MAYV E3 antibody. PNGase F digestion resulted in an expected reduction in molecular mass of MAYV E1 and E2, consistent with the hypothesis that N141 and N262 are N-glycosylated. However, there was no observed reduction in molecular mass of E1 or E2 following Endo-Hf digestion, suggestive of little to no high mannose glycosylation at these sites. Consistent with these results analysis of glycan composition by in-line LC-MS confirmed that N141 glycoprotein (N947 on entire structural polyprotein; sMAYV) consisted solely of complex-type glycosylation, and N262 glycoprotein (N586 on sMAYV) had a mixture of predominantly complex-type glycosylation, with a minor population of high mannose and hybrid-type glycosylation (Fig. 2h). Furthermore, this analysis was able to detect that E3 glycoprotein site N6 (N271 on sMAYV) consisted of complex-type glycans. This analysis also revealed nearly full-occupancy of the three N-linked glycosylation sites. In summary, this analysis shows that the glycosylation is representative of an alphavirus.

### The comparative assessment of MAYV vaccine candidates in BABL/c mice

**Fig. 3:**
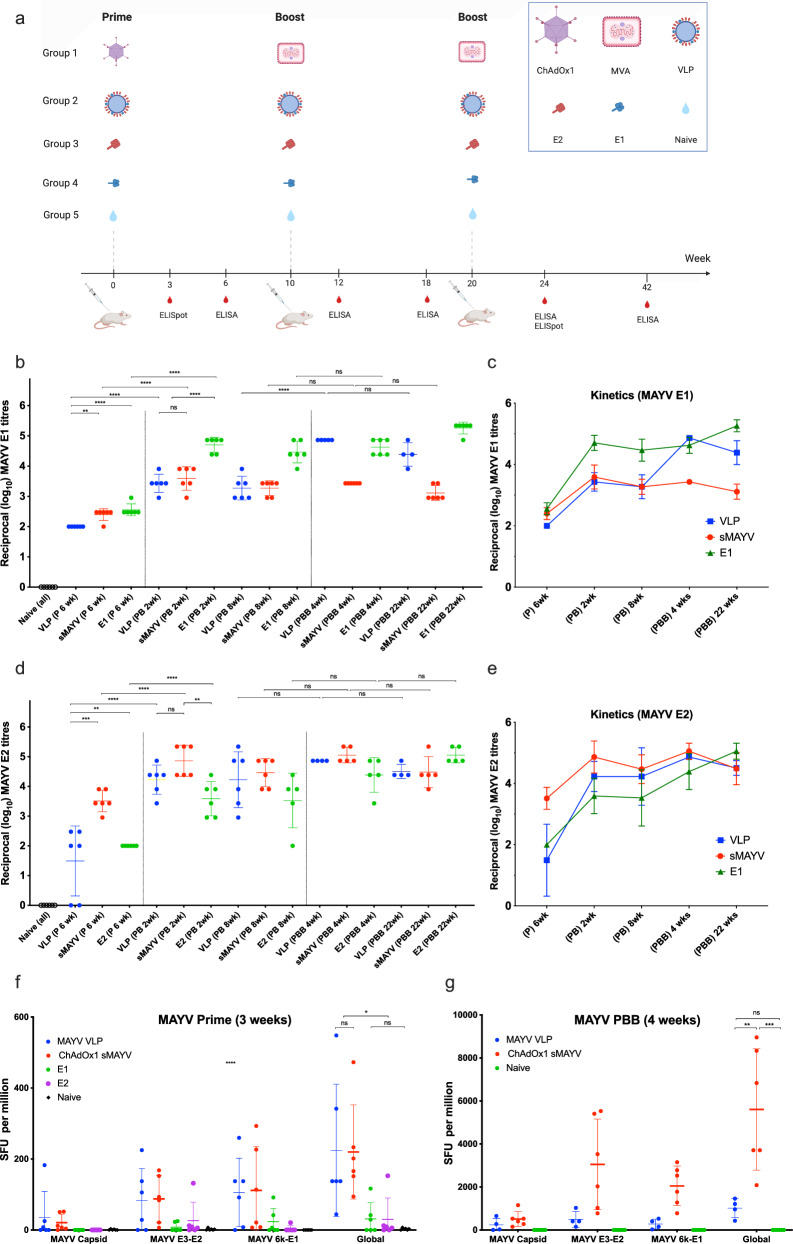
Comparative assessment of MAYV vaccine platforms in BABL/c strain of mice. **a** BALB/c mice (n = 6) were immunised intramuscularly (IM) at the intervals as outlined above and the timepoints of blood serum sampling for ELISA/ELISpot are shown. The name of the vaccination group, priming and boosting agents are shown. **b**–**e** Humoral immune responses elicited by MAYV VLP, viral-vectored vaccines and E1/E2 sub-unit vaccine. MAYV E1 or E2 ELISA was performed with sera obtained from vaccinated mice at week 6 after a single vaccination, 2 weeks and 8 weeks after P-B vaccination and 4 weeks and 22 weeks after P-B-B vaccination (**b**, **d**). Anti-MAYV E1 or E2 ELISA titres were calculated for all groups as shown in (**b**–**e**). The kinetics of anti-MAYV E1 or E2 titres over time are shown in (**c**, **e**). Turkey’s multiple comparisons test was used to analyse the groups (**b**, **d**). (**f**, **g**) Cellular immune responses elicited by MAYV VLP, viral-vectored vaccines and E1/E2 sub-unit vaccines. Peripheral blood mononuclear cells (PBMCs) were cultured with a peptide pool containing the MAYV structural antigens by ELISpot for IFNγ producing cells using mice serum; (**f**) 3 weeks post-prime (**g**) 4 weeks post-prime-boost-boost (PBB) vaccinations. Values represent the spot-forming cells (SFC) per million PBMCs. 20-mer peptides spanning the MAYV structural polyprotein (10 µg/mL) were used for stimulation. *P* values in were determined by one-way ANOVA and Tukey’s multiple comparisons test. Line colours represent mice vaccinated with each vaccine. The mean antibody titres or SFCs and error bars are shown by solid colour lines.

We have previously developed viral-vectored vaccines against MAYV and shown that ChAdOx1 May provided full protection against MAYV challenge in A129 mice with induction of highly neutralising antibody titres and MVA May was effective boosting strategy following prime vaccination with ChAdOx1 May^34,35^. Studies have demonstrated heterologous prime-boost immunisation of adenoviral and MVA vectors induce strong and long-lasting multifunctional T cell responses against malaria but this has not yet been demonstrated against MAYV^54–56^. To evaluate the immunogenicity of MAYV VLP in comparison to viral-vectored vaccines and protein sub-unit vaccines and to investigate the ability of various MAYV vaccine platforms to induce of strong and long-lasting immune responses, BALB/c mice (n = 6) were immunised intramuscularly with MAYV VLP, viral-vectored sMAYV (ChAdOx1/MVA May), MAYV E2 and MAYV E1 antigens in a non-adjuvanted prime-boost-boost regimen (Fig. 3a). The immunogenicity of MAYV vaccine candidates utilising various platforms was assessed by monitoring of anti-MAYV E2 and anti-MAYV E1 titres by ELISA at different time points following prime (P), prime-boost (P-B) and prime-boost-boost (P-B-B) immunisations. As the immunogenicity of protein-based vaccines (VLP and E1 or E2) are mainly humoral, ELISpot assays were used to monitor T cell responses at 2 time points only (3 weeks post-prime and 4 weeks post P-B-B) for confirmation.

### Immunogenicity of MAYV vaccines after immunisations

To assess the humoral response, ELISA assays were performed by coating 96-well plates with MAYV E1 or MAYV E2 to monitor the levels of anti-MAYV E1 and E2 in mice after immunisations respectively (Fig. 3b–e). At 6 weeks post-prime immunisation, the anti-MAYV E1 titres were detected in all vaccine candidates; mean titre values of VLP, ChAdOx1 sMAYV and E1 sub-units were 2.00, 2.40 and 2.55 respectively. At 2 weeks following prime-boost (P-B) immunisation, the mean antibody titres of VLP, ChAdOx1 sMAYV and E1 sub-units increased significantly to 3.43, 3.59 and 4.70 followed by a slight decline in antibody titres in all groups (3.27, 3.27 and 4.47) at 8 weeks PB (Fig. 3b). The mean antibody titres of VLP increased significantly to 4.86 while ChAdOx1 sMAYV and E1 sub-units did not have significant increase in antibody titres at 4 weeks after prime-boost-boost (P-B-B) immunisation. Interestingly, the levels of anti-MAYV E1 titres for viral-vectored group (Group 1) were lowest which may suggest that the MVA sMAYV induce only small amount of anti-E1 antibodies. The anti-E1 titres remained high (4.38, 3.11, 5.26) in all groups even after 22 weeks of PBB immunisation. The kinetics of anti-E1 titres over time is shown in Fig. 3c which highlights that the mean antibody titres for VLP and E1 based vaccines had mean antibody titres >4.00 at all time points after P-B immunisations while viral vectored vaccines were maintained >3.00 after P-B and P-B-B immunisation.

The anti-MAYV E2 titres were measured in the same manner which showed that ChAdOx1 sMAYV was the best at inducing anti-E2 antibodies post prime immunisation with mean titre of 3.51 post-prime 6 weeks (Fig. 3d). The mean titres for VLP and E2 were 1.49 and 2.00 after 6 weeks post-prime immunisation. However, upon the boost, the mean antibody titres for all groups increased significantly and maintained at high levels 8 weeks after P-B with no statistical difference in the mean titres between the groups. These mean antibody titres were further increased by second boost which maintained at high levels >4.40 for all groups at 22 weeks after P-B-B immunisation (Fig. 3e). These results suggest that the viral-vectored platform is best at inducing the anti-E2 responses early which may be important in protective efficacy against MAYV infections, but VLP and sub-unit vaccines reach similar antibody titres upon boost immunisation.

Cellular responses elicited by MAYV vaccine candidates after 3 weeks post-immunisation and 4 weeks post-prime-boost-boost (P-B-B) immunisations were assessed by interferon-γ (IFNγ) enzyme-linked immunospot (ELISpot) assay using PBMCs (Fig. 3f, g). At 3 weeks post-prime, BALB/c mice receiving ChAdOx1 sMAYV and MAYV VLP induced modest IFNγ-producing global T cell responses (mean values of 220 and 224 SFU/10^6^ PBMCs respectively), while the protein based sub-unit vaccine candidates (E1 and E2) induced minimal amount of IFNγ-producing T cell responses (mean values close to 30 SFU/10^6^ PBMCs) as expected (Fig. 3f). At 4 weeks post P-B-B immunisation, we assessed the cellular responses for the viral-vectored and VLP groups (Fig. 3g). The viral-vectored group showed high T-cell responses after stimulation with the sMAYV peptide pool (mean global T-cell responses = 5604 SFU/10^6^ PBMCs) while MAYV VLP group showed good level of T-cell response (mean global T- cell responses = 1013 SFU/10^6^ PBMCs). The majority of T-cell responses were directed towards the MAYV E3-E2 peptide pool (mean = 3048 SFU/10^6^ PBMCs) and MAYV 6K-E1 peptide pool (mean = 2048 SFU/10^6^ PBMCs) with smaller responses contributed by MAYV capsid region (mean = 507 SFU/10^6^ PBMCs) in mice vaccinated with viral-vectored vaccines. A similar trend was observed for the MAYV VLP vaccinated group with much lower T-cell responses.

### MAYV-neutralising capacity in vaccinated mice sera

**Fig. 4:**
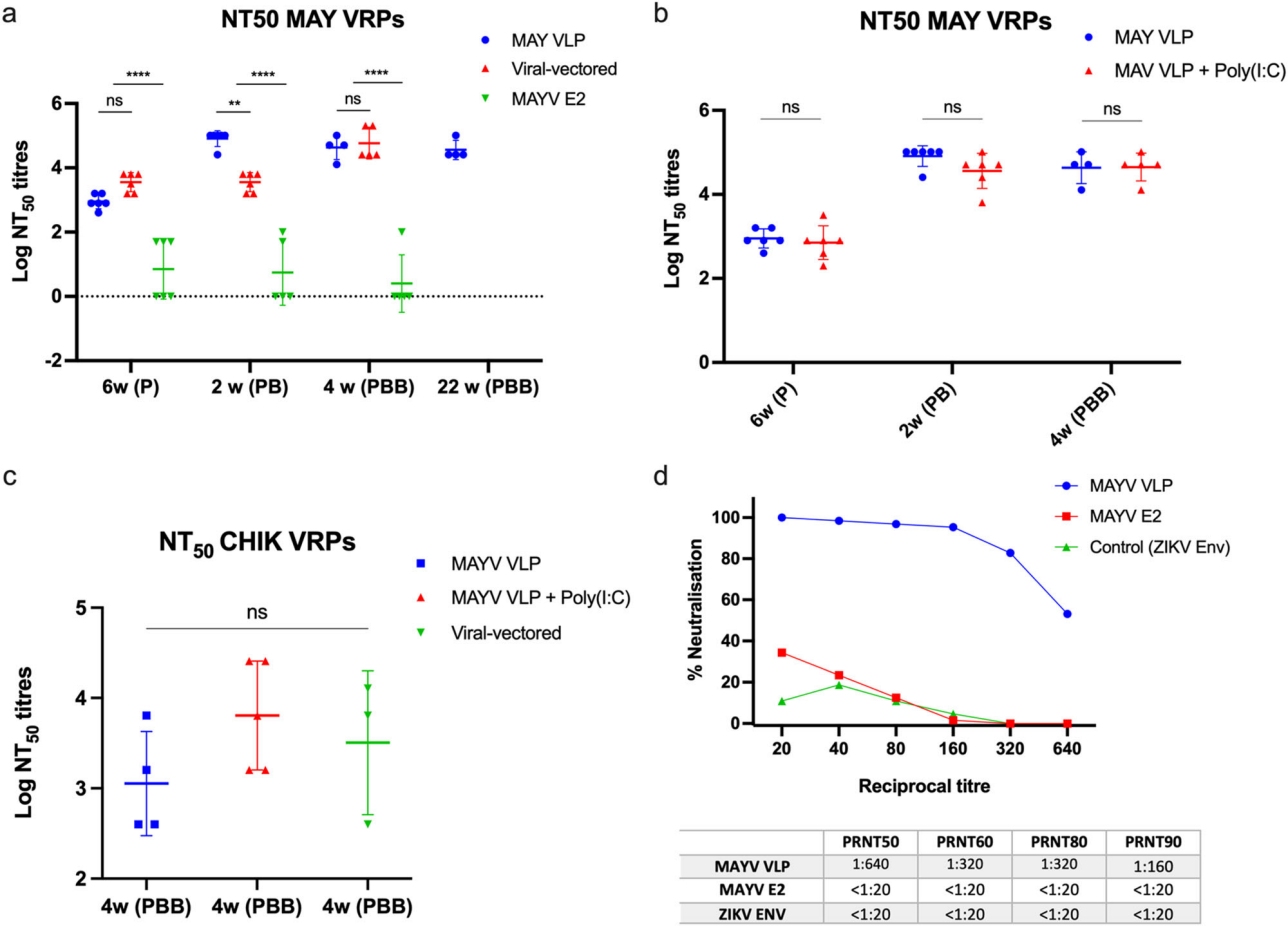
Neutralisation potency of MAYV VLP vaccinated mice sera. **a**–**c** MAYV and CHIKV VRP neutralisation assays. MAYV NT_50_ titres for BALB/c mice serum samples post immunisations with MAYV VLP, viral-vectored sMAYV or MAYV E2 are shown. **d** PRNT assay showing percentage neutralisation of mice serum vaccinated with MAYV VLP, MAYV E2 or control (ZIKV Envelope) 4 weeks post PBB. PRNT50, 60, 80 and 90 titres for each group are shown in the table. Coloured lines represent the mean with SD and error bars. *P* values in (**b**, **c**) were determined by one-way ANOVA and Tukey’s multiple comparisons test. *p* > 0.05 (ns), *p* < 0.01 (**), *p* < 0.0001 (****). *P* value in (**c**) was determined by the Mann-Whitney U test. *p* > 0.05 (ns).

We have previously reported a MAYV neutralisation assay based on chimeric MAYV virus replicon particles (VRPs) and this was used to assess neutralising capacity of our viral-vectored vaccines in a prime (ChAdOx1 or 2) or prime-boost regimen (ChAdOx-MVA)^35^. In this study, we have accessed neutralising capacity of our MAYV VLP in comparison to viral-vectored vaccines (ChAdOx1/MVA/MVA) and E2 subunit vaccines. We determined the NT50 values for BALB/c mice sera obtained after vaccination with MAYV VLP, viral-vectors (ChAdOx1-MVA-MVA) and E2 subunits at prime (6 weeks), prime-boost (2 weeks) and prime-boost-boost (4 weeks) timepoints. At 6 weeks post-prime immunisation, sera from the MAYV VLP and ChAdOx1 sMAYV vaccinated mice demonstrated high neutralisation titres against MAYV VRPs with reciprocal log NT50 titres of 2.95 and 3.55, respectively (Fig. 4a) which were significantly higher than mice vaccinated with MAYV E2 (mean NT50 = 0.85) where 3 out of 6 mice had detectable NT50 titres. Following a boost, the NT50 titres for MAYV VLP group further increased to 4.91 while viral-vectored (ChAdOx1-MVA sMAYV) and MAYV E2 groups did not have significant increase in NT50 titres (mean NT50 titres = 3.55 for viral-vectored vaccine group). After 4 weeks prime-boost-boost immunisations, the mean NT50 titres for MAYV VLP did not increase any further (mean NT50 titres = 4.63) while the NT50 titre values for viral-vectored (ChAdOx1-MVA-MVA) group increased to 4.77. Notably, MAYV E2 group failed to induce more neutralising antibodies shown by decline in NT50 titres despite the second boost with MAYV E2. To assess the durability of neutralising antibodies induced by MAYV VLP vaccination, mice sera after 22 weeks post-PBB vaccination of MAYV VLP were tested which demonstrated the mean NT50 titres remained high (mean NT50 tires = 4.56).

Next, we tested if higher neutralising antibodies can be induced by adjuvanted MAYV VLP. The poly(I:C) adjuvant was used in this study as it was previously shown to be an effective adjuvant for inducing higher antibody titres for circumsporozite protein (CSP)-based vaccine against malaria in non-human primates (NHP)^57^. We determined the NT50 values for BALB/c mice sera obtained after vaccination with MAYV VLP + Poly (I:C) adjuvants at the same timepoints (prime 6 weeks, prime-boost 2 weeks and prime-boost-boost 4 weeks) to allow the comparison to the NT50 titres determined by vaccinating MAYV VLP on its own (Fig. 4b). Interestingly, the NT50 titres for both groups were very similar with no statistical difference at all timepoints which suggest that MAYV VLP is able to high NT50 titres without use of Poly (I:C) adjuvant. Next, to assess its ability to provide cross-neutralisation against CHIKV which is a related arthritogenic alphavirus, a CHIKV VRP neutralisation assay was performed^58^ using the mice sera obtained after 4 weeks post-PBB vaccination of MAYV VLP, MAYV VLP + Poly(I:C) and viral-vectored vaccines (ChAdOx1-MVA-MVA). MAYV VLP and viral-vectored groups showed high mean NT50 titres of 3.05 and 3.51 respectively while MAYV VLP + Poly (I:C) adjuvant had higher mean NT50 titres of 3.81 but there was no statistical difference between them (Fig. 4c). These results suggest that MAYV VLP vaccines can, upon a heterologous prime-boost-boost regimen, induce high titres of cross-neutralising antibodies against CHIKV which may be further increased by use of an adjuvant. To further confirm the neutralising capacity of MAYV VLP and E2 sub-unit vaccine, the classical plaque reduction neutralisation test (PRNT) against MAYV was performed using a pool of mice sera obtained 4 weeks after PBB vaccination. Sera from MAYV VLP vaccinated mice (PBB) was also shown to have highly neutralising antibodies against MAYV with titres of 1:320 at PRNT_80_ and 1:640 at PRNT_50_ (Fig. 4d). On the other hand, sera from MAYV E2 vaccinated mice (PBB) showed a small degree of neutralisation (Fig. 4d) but had antibody titres of less than 20. The group vaccinated with control group (ZIKV E) also had <1:20. Taken together, our results confirm that our MAYV VLP vaccine can elicit functional neutralising antibodies against MAYV and induce cross-neutralising antibodies against CHIKV which is comparable to our viral-vectored vaccines.

### The protective efficacy of MAYV VLP in mouse model of MAYV infection

**Fig. 5:**
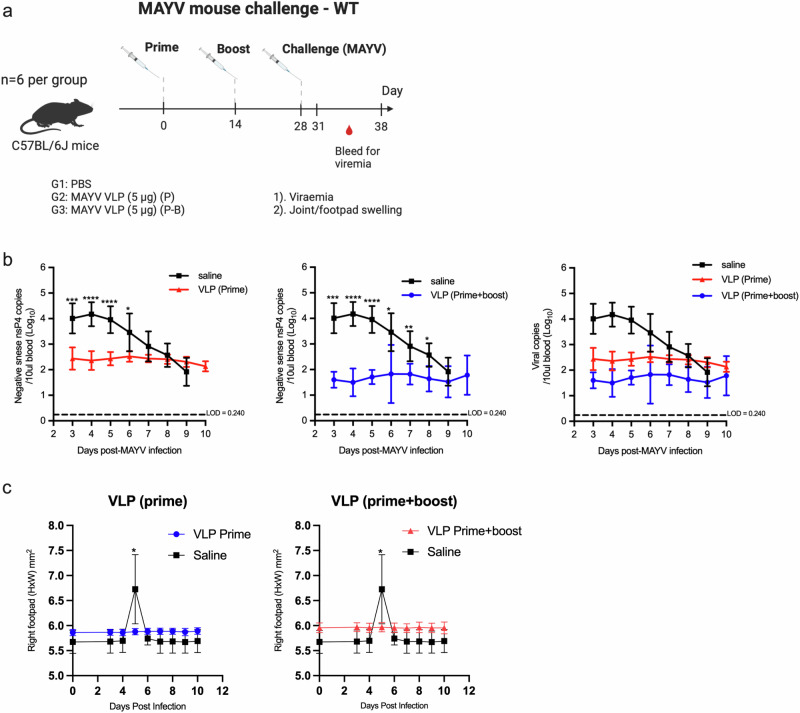
Assessment of protective efficacy induced by MAYV VLP vaccines. **a** MAYV VLP (either prime only or prime boost) vaccinated C57BL/6 mice were inoculated subcutaneously in the right hind footpad with 5 × 10^6^ PFU MAYV at 4 weeks post-immunisation. After MAYV challenge, viraemia was monitored daily from 3-day post-infection (dpi) until 10 dpi. Joint swelling of the footpad was scored starting at day 0 and after from 3 to 10 dpi. **b**. viral load kinetics of MAYV challenge groups were monitored to assess the protective efficacy. Graphs show days post-challenge on the x-axis versus viral loads on the y-axis for VLP (Prime), VLP (Prime-boost) and combined (Prime/Prime-boost). **c** Right footpad swelling of MAYV challenge groups were monitored to assess protective efficacy. Data represented as mean and SD. P-values were determined by using unpaired T-test. Data represented as mean and SD. P-values were determined by using unpaired T-test, *p < 0.05, **p < 0.01, ****p < 0.0001.

To assess the protective efficacy of our MAYV VLP vaccines, C57BL/6 mice at 4-weeks post-immunisation (n = 6) were subcutaneously challenged 5 × 10^6^ PFU MAYV (BeAr20290 strain) (Fig. 5a). Viremia and swelling at the inoculation sites were monitored as these are important hallmarks of arthritogenic alphavirus infection. Viral copies for PBS-infected group were 4.17 log_10_ copies/10µl blood on day 3–5 days post-MAYV infection when there was peak viremia (Fig. 5b). On the other hand, the mean peak viremia on day 3–5 for MAYV VLP (prime) and MAYV VLP (prime boost) vaccinated groups were 2.44 and 1.71 respectively. This suggests that vaccination with either single or two doses of non-adjuvanted 5 µg of MAYV VLP can provide statistically significant protection against viremia. In addition, a single dose or two doses of MAYV VLPs were able to provide protection against MAYV-induced foot swelling as shown by statistically significant reduction in foot swelling on day 5 compared to the PBS-vaccinated (Fig. 5c).

## Discussion

In this study, MAYV virus-like particles (VLPs) have been produced in mammalian cells for head-to-head comparison of its immunogenicity with viral-vectored vaccines and subunit immunogen in mice. The expression of the MAYV structural cassette (C, E3, E2, 6K, E1) resulted in production of secreted VLPs, which are morphologically similar to native virions as demonstrated by single particle Cryo-EM analysis of purified MAYV VLPs. Production of CHIKV VLP has been reported in multiple expression systems including mammalian, insect and yeast cells^37,59,60^. In each of these expression systems, they were shown to be highly immunogenic and provided protect against CHIKV infection in different animal models of CHIKV-induced disease^37,59,60^. Mammalian (HEK) cell-derived CHIKV VLPs have recently completed phase 2 and 3 trials and were shown to be safe, immunogenic and well tolerated in healthy volunteers^24,25^^,61^. Thus, mammalian cell-derived MAYV VLPs produced in this study were evaluated as a potential vaccine candidate and compared with the ChAdOx1/MVA sMAYV and protein-based subunit vaccines (E1 or E2). The ChAdOx1 sMAYV vaccine is based on chimpanzee adenoviral vector encoding the structural proteins, which was shown to be highly immunogenic and provided full protection against MAYV challenge in A129 mice (IFNAR -/-) with high titres of neutralising antibodies^34^.

To demonstrate that our MAYV VLPs closely resemble the native virion and thus can serve as a model for vaccine development, the structure of MAYV VLPs was determined by Cryo-EM single particle three-dimensional reconstruction. The MAYV VLP structure was found to be highly similar to the live MAYV virion structure published earlier^52^ showing the characteristic T = 4 arrangement of the 80 glycoprotein trimers made of E1 and E2 glycoproteins in addition to the capsid protein layer. It presented typical alphavirus features and organisation, which have been demonstrated previously by Cryo-EM structural studies^17,41,46,47,52,62–64^. Analysis of N-linked glycosylation of MAYV VLP revealed that sites E947 and N271, from the E1 and E3 glycoproteins, respectively, consisted of complex-type glycans, while E2 glycoprotein site N586 had mixture of oligomannose, hybrid and complex-type glycans. Interestingly, although the relative proportions appear to vary, this glycan composition seems to largely match that observed for other alphaviruses, such as SFV and EEEV where E2 glycoprotein contained conserved under-processed oligomannose-type glycans while E1 contained predominantly highly processed glycans such as complex-type and paucimannose-type glycosylations suggesting that MAYV VLPs may present glycosylation that is representative of the native virus^63,65,66^. Combined with the high level of structural resemblance of the envelope glycoproteins, these combined data indicate that MAYV VLPs present an antigenic surface closely resembling that of the native virus.

The protective efficacy of our mammalian-cell derived MAYV VLP in a prime or prime-boost regimen was assessed in C57BL/6 mice challenge with BeAr20290 strain and it was shown that vaccination with either single or two doses of non-adjuvanted MAYV VLP provide significant protection against viremia and MAYV-induced foot swelling. Our data is consistent with the recent study by Abbo et al. that showed prime-boost vaccination with 1 µg VLPs from insect cells and mammalian cells provided complete protection against adult wild-type mice (C57BL/6J) against viremia and joint inflammation after MAYV challenge and induced potently neutralising antibodies^32^. Interestingly, this study showed that the mammalian cell-derived VLPs induced higher mean neutralising antibody titres than those produced in insect cells, and this was suggested to be due to difference in glycans. A recent study identified that differential immune responses were dependent on N-linked glycosylation on alphavirus E2 glycoproteins^67^. As mosquito-derived MAYV VLP may feature mosquito-specific glycosylations such as paucimannose, the antibody specificity of MAYV VLP may be affected by E1/E2 site specific glycosylation. In this study, we have demonstrated that our mammalian cell-derived VLPs has similar structure to the live MAYV virion by Cryo-EM and the N-glycosylation pattern representative of an alphavirus by N-glycosylation analysis providing a novel insight into the MAYV VLP.

In this study, we assessed the immunogenicity of MAYV VLP in comparison to viral-vectored vaccines and protein sub-unit vaccines to investigate the ability of various MAYV vaccine platforms to induce strong and long-lasting immune responses. We have previously shown that our adenoviral vaccine (ChAdOx1 May) was able to provide protective efficacy against MAYV challenge and MVA May was effective boosting strategy following prime vaccination with ChAdOx1 May to induce highly neutralising antibodies^35^. Previous studies also demonstrated that heterologous prime-boost immunisation of adenoviral and MVA vectors induced strong and durable immune responses against malaria^54–56^. To assess if we can induce stronger and durable immune responses, heterologous prime-boost-boost immunisation of adenoviral and MVA vectored vaccination approach was chosen in this study. To allow monitoring of anti-E1 and anti-E2 titres of MAYV, MAYV E1 or E2 were produced in mammalian cells and verified by SDS-PAGE and WB using anti-His Ab and vaccinated mice serum. The humoral immunogenicity results indicate that the levels of anti-E1 and anti-E2 antibodies elicited by MAYV VLPs post-prime immunisation were lower than viral vectored vaccines such as ChAdOx1 sMAYV. This may be due to the ability of ChAdOx1 to induce strong humoral and cell-mediated immune responses and lower doses of VLPs used for initial prime vaccination (mice were primed with 2 µg and boosted with 5 µg of VLP). Previous studies in mice involved subcutaneous immunisations of CHIKV VLP at 10, 20 and 40 µg with an adjuvant for inducing neutralising antibodies^37^. It will be interesting to test if the use of a higher dose of VLP will increase the immunogenicity and efficacy of MAYV VLPs similar to ChAdOx1 sMAYV after prime immunisation. T cell immunogenicity elicited by ChAdOx1/MVA sMAYV vaccines were significantly higher than that of VLPs. Upon prime-boost or prime-boost-boost immunisations, both anti-E1 and E2 antibody titres of MAYV VLP were comparable to viral vectored vaccines and sub-unit vaccines. In a PRNT assay against MAYV, mice serum immunised with VLPs (P-B-B) produced high titres of neutralising antibodies, while that of mice immunised with isolated MAYV E2 did not have detectable titres of neutralising antibodies. MAYV VRP-based neutralisation was used to assess further neutralising capacity of our MAYV VLP in comparison to viral-vectored vaccines (ChAdOx1/MVA/MVA) and E2 subunit vaccines. It was shown that MAYV VLP was able to induce high neutralisation titres comparable to ChAdOx1 sMAYV following a single non-adjuvanted immunisation, which significantly increased upon a boost and the neutralising titres remained high even after 22 weeks post-PBBB immunisation while MAYV E2 subunit vaccines failed to induce detectable titres of neutralising antibodies. The potential effect of use of Poly (I:C) adjuvant for MAYV VLP vaccines was assessed and showed similar MAYV neutralising antibody titres and higher cross-neutralising antibodies against CHIKV compared to immunisation of MAYV VLP on its own. These results demonstrate that MAYV VLP can induce high titres of MAYV-specific neutralising antibodies and cross-neutralising antibodies against CHIKV upon boosting which may be further increased by use of an adjuvant. The reason for lack of neutralising antibodies in the MAYV E2 sub-unit vaccine could be due to absence of adjuvant to boost the immune responses or the absence of conformational epitopes, which may be essential in providing the protective efficacy. Previous studies comparing the immunogenicity and protective efficacy of the CHIKV E1 and E2 subunits, and the CHIKV VLP in AG129 mice, have shown that upon prime-boost immunisations subcutaneously with Maxtrix M adjuvant, CHIKV VLP induced high neutralising antibody titres^38^. In addition, CHIKV VLP provided complete protection against lethal CHIKV challenge in mice whereas CHIKV E1 and E2 resulted in lower levels of neutralising antibodies with partial protection^38^. As ChAdOx1 sCHIKV was shown to assemble into VLP for CHIKV^68^, MAYV VLP could be expected to form VLPs by immunisation of ChAdOx1 sMAYV vaccines. Therefore, viral-vectored (ChAdOx1/MVA May) vaccines and VLPs would induce conformational epitopes, which might be physiologically relevant to confer protective efficacy in the challenge studies. We have demonstrated in this study that expression of the transgene in our viral-vectored vaccines in mammalian cells leads to the assembly of the MAYV VLP which has been shown to be structurally similar to the live MAYV virion. Further studies with MAYV VLP and viral-vectored MAYV vaccines using MAYV challenge models would inform on whether these vaccines can provide protective efficacy in animal models, including non-human primates and provide a potential new vaccine for MAYV epidemic preparedness.

## Materials and methods

### Design and cloning of MAYV structural transgene

The structural polyprotein sequence of MAYV was synthetically generated by GeneArt following codon optimisation for efficient expression in mammalian cells and inclusion of a Kozak sequence to improve the initiation of translation as previously described^34^. To clone the transgenes into the pHLsec expression plasmid without its secretory signal, an EcoRI site was included in the forward primer and an Acc65I restriction site was inserted into the reverse primer with a stop codon to prevent expression of His-tag in the pHLsec plasmid^39^. The plasmids containing sMAYV with new restrictions sites were PCR-cloned using AccuPrime^TM^ Pfx SuperMix (Invitrogen^TM^) and subsequently ligated into pHLsec expression plasmid. The recombinant DNA plasmids were purified from *E. coli* using the miniprep kit (Qiagen, Hilden, Germany) and the content of each plasmid was verified by restriction analysis and DNA sequencing (Source, BioScience).

Forward primer (CTGAAGAATTCGCCACCATGGATTTTCTGCC)

reverse primer (GCTTGGGTACCTCATCATCTTCTCAGGGTGATACAG)

### Expression and purification of MAYV VLP from mammalian cells

The large-scale transfection of MAYV VLP was carried out in roller bottles using the standard PEI transfection of HEK293T cells as previously described^69^. Briefly, HEK293T cells were grown in DMEM (Sigma–Aldrich, UK) supplemented with 10% FCS, 1 mM glutamine and 1× non-essential amino acids in roller bottles (Greiner Bio One, UK) at 37 °C for 3–4 days at 30 rpm until the cells reach ~90% confluency. The standard transfection was performed by incubating 0.5 mg of plasmid DNA with 1 ml of 1 mg/ml polyethylene-imine (PEI) for 10 min in a final volume of 50 ml serum free growth media per each roller bottle. The mixture was then added to the cells that contained 200 ml of fresh growth media and incubated at 37 °C with the bottle rotating at 30 rpm for 4–5 days. The supernatant was harvested by centrifuging at 3000 × *g* for 20 min to remove cell debris, and filtering through a 0.22-μm Stericup Filter (Millipore, UK). Secreted protein fractions were precipitated by using PEG virus precipitation kit (Abcam, ab 102538) according to the protocol from the manufacturer and loaded on a sucrose gradient 20–60% prepared by gradient master (BioComp Instruments). Sucrose gradients were centrifuged at 28,000 rpm (SW40 Ti rotor, Beckman) for 16 h at 4 °C. The white band containing VLPs was isolated and resuspended in 10 ml STE buffer. The VLPs were pelleted by centrifugation through 20% sucrose cushion with 30,000 rpm for 2 h at 4 °C. The pellet was resuspended in STE buffer and was assessed for the presence of VLP proteins by SDS-PAGE and western blotting analysis. The integrity of VLPs was checked by transmission electron microscopy. The VLPs were stored at –80°C for future use.

### Characterisation of VLP by SDS-PAGE and western blot

The protein composition of purified MAYV VLPs was analysed by SDS-PAGE and Western blot (WB) using 1:500 dilutions of mice sera following a vaccination with ChAdOx1 May (sMAYV) using previous described protocol^35^. For Coomassie Blue stain, the gel was stained with Instant Blue Coomassie stain for 1 h and de-stained in dH_2_O before imaging. For Western blot (WB), proteins were transferred onto nitrocellulose membranes (Bio-Rad Trans-Blot® TurboT^M^). Membranes were blocked with 5% skim milk in 0.1% PBS/T for 1 h and then incubated with 1:500 dilutions of mice sera following a vaccination with ChAdOx1 May (sMAYV) or Chik (sCHIKV). After washing with PBS/T, membranes were incubated for 1 h with an HRP-conjugated goat anti-mouse IgG (Bio-Rad Cat. 170-6516). Finally, membranes were washed again using PBS/T and incubated with a chemiluminescent substrate (Clarity^TM^ Western ECL Blotting Substrates, BIO-RAD); signal was detected using a chemiluminescent Western blot imaging system (Image Lab, Bio-Rad).

### Transmission Electron microscopy (TEM)

Formvar/carbon 200 Mesh Cu grids were glow-discharged in air and loaded with 3.5 μl of sample of MAYV VLP. Excess liquid was removed, and the grids were washed 3 times with MilliQ water. Finally, grids were treated with 2% uranyl acetate for 30 s, excess uranyl acetate was carefully removed using filter paper. The grids were air-dried and analysed with a T12 transmission electron microscope (FEI, Eindhoven, The Netherlands).

### Glycan analysis by western blotting

To determine the glycosylation of MAYV VLP and to understand viral biology and vaccine design, purified VLP sample was treated with PNGase F (New England Biolabs) and EndoF1-GST to determine their glycosylation status according to the protocol from NEB. Briefly, the samples were treated with 1 µl glycoprotein denaturing buffer (10×) in 9 µl of dH_2_O for 10 min at 95 °C. The denatured proteins were made into a total reaction volume of 20 µl by adding 2 µl GlycoBuffer 2 (10X), 2 µl 10% NP-40 buffer, 6 µl dH_2_O and 1 µl PNGase F or EndoH-GST and incubating for 1 h at 37 °C. Treated and non-treated protein samples were analysed by SDS-PAGE and WB.

### Cryo-EM data collection and data processing

An aliquot (3.5–4.5 μl) of purified VLP samples (diluted to ~1–2 mg/ mL in STE buffer) was applied to glow discharged EM grids [UltraAuFoil ® Holey Gold (Quantifoil) or Lacey carbon grid] and vitrified by plunge-freezing into liquid ethane using Vitrobot mark IV (Thermo Fisher Scientific) operated at 4°C, 100% relative humidity. The integrity of plunge-frozen cryo-EM grids and the quality of VLP samples were assessed by screening the grids using a 200-kV transmission microscope (Glacios, Thermo Fisher Scientific). Cryo-EM data were acquired on a 300-kV Titan Krios (Thermo Fisher Scientific) equipped with an energy filter (GIF quantum LS; Gatan; zero-loss mode with 20-eV slit width) and a direct electron detector (K2 summit; Gatan). A total of 9234 movie stacks (30 frames) were collected in electron-counting mode at dose rate of 〜1 e^−^ Å^−2^ s^−1^ with a calibrated magnification of 50,000× and the defocus ranges of −1.5 to −2.5 µm, resulting in a total dose of 30 e^−^ Å^−2^ and corresponding pixel size of 1.34 Å. Data processing was performed within the Scipion software. Movie frames were aligned and averaged with dose weighting in MotionCor2 to compensate for specimen drift and reduce the effects of electron beam-induced specimen damage. The contrast transfer function (CTF) parameters were estimated locally using CTFIND. Particles were picked from averaged images automatically using Xmipp3 auto-picking in Scipion. 32,989 particles were picked out initially and 26,857 particles were finally retained after the 2D classification for the subsequent 3D auto-refine in Relion 3.1 using a box size of 800 pixels and with the icosahedral (I1) symmetry applied. After two iterations of particle-based CTF refinement and particle polishing, the final resolution was 4.5 Å based on the Fourier shell correlation at 0.143 criterion.

### Glycopeptide analysis by nano LC-MS

MAYV VLPs were proteolytically digested with trypsin (Promega) and chymotrypsin. Reaction mixtures were dried and peptides/glycopeptides were extracted using C18 Zip-tip (MerckMilipore) following the manufacturer’s protocol. Samples were resuspended in 0.1% formic acid prior to analysis by liquid chromatography-mass spectrometry using an Easy-nLC 1200 system coupled to an Orbitrap Fusion mass spectrometer (Thermo Fisher Scientific). Glycopeptides were separated using an EasySpray PepMap RSLC C18 column (75 μm × 75 cm) with a 240-min linear solvent gradient of 0–32% acetonitrile in 0.1% formic acid, followed by 35 min of 80% acetonitrile in 0.1% formic acid. Other settings include an LC flow rate of 200 nL/min, spray voltage of 2.8 kV, capillary temperature of 275 °C and an HCD collision energy of 50%. Precursor and fragmentation detection were performed using an Orbitrap at the following resolution: MS1 = 100,000 and MS2 = 30,000. The automatic gain control (AGC) targets were MS1 = 4e5 and MS2 = 5e4, and injection times were MS1 = 50 and MS2 = 54. Glycopeptide fragmentation data were extracted from raw files using ByonicTM (Version 3.5.0) and ByologicTM (Version 3.5-15; Protein Metrics Inc.). Glycopeptide fragmentation data were manually verified. The extracted ion chromatographic areas for each true-positive glycopeptide, with the same amino-acid sequence, were compared to determine the relative quantitation of glycoforms at each specific N-linked glycan site.

### Viral-vectored vaccine production

ChAdOx1 and MVA encoding sMAYV (C, E3, E2, 6K, E1) were produced as previously described^34,35^. Briefly, to generate ChAdOx1 vaccines, the shuttle plasmids containing attL regions sequences were each recombined with those attR regions contained in the destination vector ChAdOx1 using an in vitro Gateway reaction (LR Clonase II system, InvitrogenTM, Carlsbad, CA, USA). Successfully recombined ChAdOx1 sMAYV plasmids were verified by DNA sequencing using flanking primers. Standard cell biology and virology techniques were performed to generate the non-replicative adenoviral vectors^70^. To generate MVA based vaccines, the sMAYV cassette was digested with KpnI and XhoI to allow in-frame ligation between the P7.5 promoter and the TKR locus, contained in the entry plasmid MVA. Ligated DNA plasmids were expanded in *E. coli* and a midiprep (Qiagen) was used for plasmid purifications. Resulting plasmids were verified by restriction analysis and 5∲ and 3∲ flanking sequencing, and co-transfected to produce MVA sMAYV using the methodology as previously described^71^.

### Production and purification of MAYV E1/E2 antigens

The expression of MAYV E1/E2 antigens were carried out in roller bottles using the standard PEI transfection of HEK293T cells as previously described^34,35,68,72^.

### Animals and immunisation

Female inbred BALB/c (H-2d), (6–8 weeks) were used for the assessment of immunogenicity (*n* = 6 mice per group). Mice were purchased from Envigo. All animals and procedures were used in accordance with the terms of the UK Home Office Animals Act Project License. Procedures were approved by the University of Oxford Animal Care and Ethical Review Committee (PPL 30/2414). Mice were intramuscularly immunised with ChAdOx1 sMAYV at a dose of 1 × 10^8^ IU as prime and boosted twice with Modified Vaccinia Ankara virus (MVA) encoding sMAYV at a concentration of 1 × 10^7^ pfu per ml as previously described^68,73^. All viral vector vaccines were prepared in endotoxin-free PBS and administered intramuscularly into right or left thigh muscle. All recombinant protein vaccinations were administered intramuscularly as a 50 µl dose containing sterile PBS and 3 µg (prime) or 10 µg (boost) of protein. For VLP vaccinations, 50 µl vaccine dose comprised PBS and 2 µg (prime) or 5 µg (boost) of VLP.

### Enzyme-linked immunosorbent assay

Antibody binding to the recombinant MAYV E1 or E2 antigens in mice serum was measured by an IgG ELISA as previous described^34,35,68,72^. Briefly, mice sera were diluted in Nunc Maxisorp Immuno ELISA plates coated with the MAYV E1 or E2 diluted in PBS to a final concentration of 2 µg/mL and incubated at room temperature (RT) overnight. Plates were washed 6 times with PBST and blocked with 300 µL with Pierce^TM^ protein-free (PBS) blocking buffer (Thermo Fisher Scientific, Waltham, MA, U.S.) for 2 h at RT. Mouse serum was added and serially diluted 3-fold down in PBS/T with 50 µL per well as final volume and incubated for 2 h at RT. Following washing 6 times with PBS/T, bound antibodies were detected after a 1 h incubation with 50 µL of alkaline phosphatase-conjugated antibodies specific for whole mouse IgG (A3562-5ML, Sigma–Aldrich, SLM, U.S.). Development was achieved using 100 µL of 4-nitrophenylphosphate diluted in diethanolamine buffer and the absorbance values at OD_405_ were measured and analysed using a CLARIOstar instrument (BMG Labtech, Aylesbury, GB). Serum antibody endpoint titres were defined by an absorbance value three standard deviations greater than the average OD_405_ of the control.

### Ex-Vivo IFNγ ELISpot assay

ELISpots were carried out using peripheral blood mononuclear cells (PBMCs) using 20-mer specific MAYV structural peptides overlapped by 10 a.a using previously described method^68^. Briefly, MAIP ELISpot plates (Millipore) were coated with an anti-mouse-IFN-γ mAb AN18 (Mabtech), after 1 h blocking with complete DMEM media. PBMCs were isolated from peripheral blood collected from tail vein bleeds into tubes containing 200 μl 10 mM EDTA/PBS. Erythrocytes were lysed using ACK lysis buffer and PBMCs harvested through centrifugation. Cells were washed, re-suspended in complete medium, counted using Countess Automated Cell Counter and 100 μl of media containing mouse PBMCs (at various concentrations) were plated into test wells. 50 μl of each of peptide pools diluted in media (10 μg/ml) and 5 × 10^6^ naïve splenocytes were added to test wells. Control wells with PBMCs from naïve mice without peptide and no cells with peptide were included. Plates were incubated at 37 °C, 5% CO2 for ~16 h. Plates were then washed with PBS and incubated with 50 μl biotinylated anti-mouse-IFN-γ mAb R4-6A2 (Mabtech) in PBS (1:1000) for 2 h. After washing, plates were incubated with a streptavidin alkaline phosphatase polymer (1:1000 in PBS) for 1 h. After another washing step, spots were developed by addition of colour development buffer (BIORAD). Once spots were visible, the reaction was stopped by washing the plate with water. Spots were counted using an ELISpot plate reader (AID, Germany) and expressed as spot forming units (SFU) per million of PBMCs producing IFN-γ. Background responses in media-only wells were subtracted from those measured in wells stimulated by peptide pools.

### Plaque reduction neutralisation test

PRNT assays were performed against MAYV (CH3 strain) as previously described^74^. Sera were heat-inactivated at 56 °C for 1 h; then diluted 2-fold in media after an initial 10-fold dilution. A known amount of virus was incubated with each serum dilution for 1 h, after which each virus-serum dilution was used to infect Vero cell monolayers in a 12-well plate. Plates were treated like a virus titration from that point on. The mean number of plaques in un-neutralised wells was 64, making 50% reduction (PRNT50) yield 32 plaques, 80% reduction (PRNT_80_) yield 12 plaques and 90% reduction (PRNT_90_) yield 7 plaques. The limit of detection (LOD) was a titre of 1/20 and any titre below this was set at half of the LOD (titre of 1/10)

### MAYV and CHIKV VRP-based neutralisation assay

MAYV neutralising antibody titres were determined as described previously using chimeric MAYV replicon particles (VRPs) expressing Gaussia luciferase (Gluc) based on previously developed MAYV VRP^35^. Briefly, 2 × 10^4^ BHK-21 cells were seeded in 96-well plates per well. The next day, VRPs (MOI of 5, calculated based on VRP RNA copies/ml) were preincubated with 2-fold serial dilutions of heat inactivated serum samples for 1 h at 37 °C before the mixtures were added to the 96-well plates. After incubation for 1 h at 37 °C, the inocula were removed, cells were washed with PBS and medium was added. Readout of secreted Gaussia was performed at 24 h post infection using a Renilla luciferase assay system (Promega, Southhampton, U.K.). Sera-neutralisation-capacity was determined by measuring Gluc activity and relating it to Gluc readout after VRP application without serum. Results are presented as 50% neutralisation (NT50) titres. CHIKV VRP-based neutralisation assay was used to determine the NT50 titres for CHIKV as previously described^58^.

### MAYV mouse challenge study

The virus strain MAYV BeAr20290 was isolated from infected Brazilian patients, kindly provided by Prof. José Luiz Modena (UNICAMP) and Prof. Luis Tadeu de Moraes Figueiredo (Faculdade de Medicina Ribeirão Preto, USP). MAYV was propagated and quantified by standard plaque assay in Vero CCL-81 cells as plaque forming units (PFU)^75^. A 3 group of C57BL/6 mice (n = 6 per group) were immunised in 50 µl vaccine dose comprised PBS and 5 µg (prime) and boosted with 5 µg of VLP at 2 weeks post-prime. MAYV was inoculated subcutaneously in the ventral side of the right hind footpad toward the ankle with 5 × 10^6^ PFU MAYV in 50 μl PBS. Viraemia was monitored daily from 3-day post-infection (dpi) until 10 dpi. Briefly, 10 μL of blood collected from the tail vein was diluted in 120 μL PBS and 10 μL citrate-phosphate-dextrose solution (Sigma-Aldrich). Viral RNA was extracted with E.Z.N.A. Viral RNA Kit (Omega Bio-Tek-R6874-02) in accordance with manufacturer’s protocol. To measure the levels of replicating virus in the extracted samples, RNA copies of negative sense *NsP*4 were quantified using RT-PCR (*Nsp4* Forward 5′-TACCATGTCAGATATGCTAAGCCTCGG-3′; Reverse 5′-CGCCACTGTAGGGTAGTTGCG-3′; probe 5′-FAM TCTGTGCCGGTGATGCAAAGACTTAGCAGCGC BHQ-1-3′). The reaction was normalised using Quant Studio 3 Real-Time PCR System (Thermo Fisher Scientific) and the standard curve was generated from the viral stock previously titled. Joint swelling of the footpad was scored starting at day 0 and after from 3 to 10 dpi. Using a digital caliper, the hind paw swelling outcome was done for both height (thickness) and breadth of the foot and were quantified as [height × breadth].

## Data Availability

The cryo-EM density map of MAYV VLP from this study has been deposited at the the Electron Microscopy Data Bank under code EMD-15898.
