# Author Correction: Immunogenic recombinant Mayaro virus-like particles present natively assembled glycoprotein

**DOI:** 10.1038/s41541-025-01122-z

**Published:** 2025-04-19

**Authors:** Young Chan Kim, Yasunori Watanabe, Arlen-Celina Lücke, Xiyong Song, Raquel de Oliveira Souza, Robert Stass, Sasha R. Azar, Shannan L. Rossi, Carla Claser, Beate Mareike Kümmerer, Max Crispin, Thomas A. Bowden, Juha T. Huiskonen, Arturo Reyes-Sandoval

**Affiliations:** 1 Department of Paediatrics, Oxford Vaccine Group, University of Oxford, Oxford, UK; 2 Division of Structural Biology, Wellcome Centre for Human Genetics, University of Oxford, Oxford, UK; 3 Institute of Virology, Medical Faculty, University of Bonn, Bonn, Germany; 4 Institute of Biotechnology, Helsinki Institute of Life Science HiLIFE, University of Helsinki, Helsinki, Finland; 5 Department of Parasitology, Institute of Biomedical Sciences, University of São Paulo, São Paulo, SP Brazil; 6 Department of Pathology and the Institute for Human Infection and Immunity, University of Texas Medical Branch, Galveston, TX USA; 7 Department of Microbiology, Immunology and Parasitology, Federal University of São Paulo (UNIFESP), São Paulo, SP Brazil; 8 German Centre for Infection Research (DZIF), Partner Site-Bonn-Cologne, Bonn, Germany; 9 School of Biological Sciences, University of Southampton, Southampton, UK; 10 Instituto Politécnico Nacional, IPN. Av. Luis Enrique Erro s/n. Unidad Adolfo López Mateos, Mexico City, Mexico

**Keywords:** Infectious diseases, Vaccines, Virology

Correction to: *npj Vaccines* 10.1038/s41541-024-01021-9, published online 17 December 2024

In this article the author name “Arlen-Celina Lücke” was incorrectly written as “Lücke Arlen-Celina”. The original article has been corrected.

